# Emergency Department Utilization by Race, Ethnicity, Language, and Medicaid Status

**DOI:** 10.5811/westjem.41511

**Published:** 2025-07-11

**Authors:** Daniel J. Berger, Colin Jenkins, John Wong-Castillo, Sarahrose Jonik, Nancy P. Gordon

**Affiliations:** * Virginia Commonwealth University Health System, Departments of Emergency Medicine and Internal Medicine, Richmond, Virginia; † University of California, San Francisco, Division of Critical Care Medicine, Department of Anesthesia and Perioperative Care, San Francisco, California; ‡ University of California San Francisco Fresno, Department of Emergency Medicine, Fresno, California; § Penn State College of Medicine, Hershey, Pennsylvania; || Kaiser Permanente, Division of Research, Pleasanton, California

## Abstract

**Introduction:**

Emergency department (ED) use varies by age, sex, race, ethnicity, language preference, and payor type. Most studies comparing ED use by patients with English vs non-English preference (ELP/NELP) have used racially aggregated data, potentially masking differences across population subgroups. In this study we aimed to disaggregate the associations of race, ethnicity, language preference, and Medicaid coverage with ED utilization.

**Methods:**

We used cross-sectional study electronic health record data for 2,047,105 Kaiser Permanente Northern California members who were 25 – 85 years of age in January 2019 and had been continuous health plan members during 2018 – 2019. We tabulated the percentages of adults in seven racial and ethnic groups (White, Black, Hispanic, Chinese, Filipino, Vietnamese, South Asian) within three age groups (25 – 44, 45 – 64, 65 – 85) who had ≥1 ED visit in 2019. Modified log-Poisson regression was used to examine racial, ethnic, and language preference differences after adjusting for demographic and Medicaid status covariates.

**Results:**

The study population was 51.8% White, 53.2% female, 9.6% NELP, and 6.2% Medicaid-insured. Overall, 18% had ≥ 1 ED visit. Compared with White adults, Black and Hispanic adults were more likely and Chinese, Vietnamese, and South Asian adults were less likely to have ≥ 1 ED visit. After adjusting for all covariates, NELP adults 25 – 64 years of age were 10% less likely to have had an ED visit. However, while NELP was associated with a 10–20% lower ED visit prevalence among Hispanic, Filipino, Chinese, and Vietnamese adults 25 – 64, the prevalence was 10% higher among White and South Asian adults 45 – 64 and Filipino and South Asian adults aged 65 – 85. Adults with Medicaid coverage aged 25 – 64 were twice as likely and adults aged 65 – 85 were 50% more likely to have had ≥ 1 ED visit.

**Conclusion:**

This study of a US adult health-plan membership found several significant differences in ED use across racial, ethnic, and language subgroups and a higher prevalence of ED use by Medicaid-covered adults ≤ 65 years of age in most racial and ethnic groups. Our findings highlight the importance of using disaggregated data, particularly for Asian ethnic groups, when comparing ED use in different populations. Further research is needed to identify similarities and differences in social, personal, and policy factors driving ED use in diverse adult populations to better inform population-specific health interventions.

## INTRODUCTION

### Background

In recent years, the rising use of emergency departments (ED) nationwide has highlighted challenges within the healthcare system. The increase in the number of ED visits has been contemporaneous with a national decline in access to primary care services.^1^–^2^

Previous research has identified sociodemographic and healthcare access factors associated with ED visits including age, sex, race and ethnicity, language barriers, payor and coverage status, and access to a regular primary care physician.^3^,^4^,^5^–^6^ However, many of these studies are now over a decade old and included adults who lacked health insurance and/or a usual source of primary care, factors that have been shown to increase likelihood of ED use. Additionally, most studies examining differences in ED use by adults with limited English proficiency (LEP) have compared Hispanic adults with and without LEP or compared racially aggregated populations with non-English language preference (NELP) vs English language preference (ELP), which may mask differences between and within racial and ethnic groups.^7^–^9^ Researchers have begun to advocate for using racially/ethnically disaggregated data for such comparisons to disentangle the effects of race and ethnicity from those of English or non-English language preference (as a proxy for English proficiency), as this could lead to better understanding of drivers of healthcare utilization within different demographic subgroups. In turn, this could improve efforts to develop and monitor system-level interventions aimed at reducing suboptimal healthcare utilization in higher risk segments of the population.^10^,^11^

In this study we aimed to disaggregate the associations of race and ethnicity, language preference, and Medicaid coverage with the prevalence of ED visits among younger, middle-aged, and older adults in a contemporary study population of adult, Northern California health plan members who received healthcare from the same integrated healthcare delivery system.

## METHODS

### Study Design

In this cross-sectional retrospective study we analyzed data from an existing electronic health record (EHR)-derived research dataset to compare the percentages of adults in seven racial and ethnic groups who in 2019 made ≥ 1 ED visit to a Kaiser Permanente Northern California (KPNC) medical center. This study was conducted under the scope of a broader study of racial and ethnic health and healthcare disparities that was approved by the KPNC Institutional Review Board with a waiver of informed consent and HIPAA authorization.

### Setting and Study Population

Kaiser Permanente Northern California is an integrated healthcare delivery system that provides primary and specialty outpatient and hospital care along with pharmacy and laboratory services, a 24/7 telephone Advice Call Center, a patient portal, and interpreter services to a sociodemographically diverse health plan membership that includes >3.4 million adults who mostly reside in the Greater San Francisco Bay Area, Sacramento, the Silicon Valley, and Central Valley. The KPNC adult membership includes a very low percentage of adults covered by California’s Medicaid program and is similar to the non-Medicaid insured adult population of Northern California with regard to social, demographic, and health characteristics. 12 Upon enrollment in the health plan, all members are assigned a primary care physician within the healthcare system and are encouraged to use primary care services and to obtain clinical advice using the Appointment and Advice Call Center and the patient portal.

**Population Health Research Capsule**

What do we already know about this issue?

*Emergency department (ED) use has been shown to vary by age, sex, race, ethnicity, language preference, and health insurance type*.

What was the research question?

*What demographic factors predict ED use in an insured adult population receiving care within an integrated care delivery system?*

What was the major finding of the study?

*Large differences in utilization were seen across race/ethnicity, particularly between different Asian ethnic groups, but not by language preference within ethnic groups*.

How does this improve population health?

*Our study highlights the importance of using disaggregated data, particularly for Asian ethnic groups, when exploring sociodemographic factors associated with ED use*.

We used secondary data from an existing EHR-derived research dataset that included demographic variables, Medicaid coverage status, and selected healthcare utilization data for 2.52 million adults 25 – 89 years of age who were continuous KPNC members during calendar year 2019.^13^ The study population was comprised of a subset of 2,047,105 adults 25–85 in January 2019, who had been continuous health plan members during 2018–2019. It included 1,061,066 White (0.5% NELP), 153,817 Black (0.4% NELP), 431,031 Hispanic (29.2% NELP), 133,733 Chinese (30.0% NELP), 132,247 Filipino (4.0% NELP), 97,549 South Asian (6.8% NELP), and 37,662 Vietnamese (31.9% NELP) men and women.

### Study Variables

#### Sociodemographic Characteristics

Age, sex at birth, and race and ethnicity were available for all adults from the source cohort. Detailed information about how adults were assigned to a racial/ethnic group can be found in the article describing the source dataset.^13^ Spoken language preference and Medicaid coverage status during 2019 were available for nearly all individuals in the study cohort. Language preference was collapsed into non-English language preference (NELP) vs. English language preference (ELP).

#### ED Visits

The source dataset included a variable for total number of ED visits during 2019. The *International Classification of Diseases* code indicating primary diagnosis for each ED visit was not available in the dataset. In this study, our outcome of interest was ≥1 ED visit during 2019.

### Statistical Analysis

All analyses were conducted in 2024 using SAS v9.4 (SAS Institute, Inc, Cary, NC). We calculated the unadjusted prevalence of ≥ 1 ED visit by race and ethnic group for adults in three age groups (25 – 44, 45 – 64, and 65 – 85 years of age) and used chi-square tests to identify statistically significant differences (at *P* < .05) between White and non-White racial/ethnic groups within each age group. White patients were chosen as the reference group because they comprised the largest proportion of the sample. Because the very large racial and ethnic subgroups resulted in significant *P*-values for very small differences in prevalence, we made an a priori decision that to be considered *meaningfully* significant, differences between groups needed to be ≥ 1 percentage point and have a chi-square *P*-value < .05. Unless otherwise noted, subgroup differences mentioned in the text met the criteria for meaningful difference.

We used modified log-Poisson regression models to estimate adjusted prevalence ratios (aPR) with 95% confidence intervals (CI) that compare the prevalence of ≥ 1 ED visit among Black, Hispanic, Chinese, Filipino, South Asian (adults with ethnic origins in India, Pakistan, Afghanistan, Bangladesh, Sri Lanka, Nepal, or Bhutan, or who were Fijian Indian), and Vietnamese adults to the prevalence among White adults within each age group after controlling for sex, age as a five-year interval variable, NELP/ELP status, and Medicaid coverage.^14^ Medicaid coverage served as a proxy for very low income, but additionally, Medicaid-covered members did not have a copay for ED visits, whereas ED visits for commercially and Medicare-covered adults had a substantial co-pay. Black adults were excluded from analyses comparing NELP vs. ELP status within racial/ethnic groups due to small subgroup sizes. Adults with missing data were only excluded from bivariate and multivariate analyses that included that variable. Finally, our statistical analyses did not adjust for multiple comparisons, but we report the results of all statistical tests.^15^

## RESULTS

### Demographic Characteristics of the Study Population

The overall study population of 2,047,105 was 53.2% female, 9.6% NELP, and 6.2% Medicaid-insured, with a mean age of 52.3 years of age (standard deviation 15.2 years). Descriptive data for the overall study population and the seven racial and ethnic groups by age group, are found in the Table.

### Percentages of the Study Population with ≥ 1 ED Visit in 2019

During the 2019 study period, 18.0% of the 2,047,105 patients had ≥ 1 ED visit, with adults 65 – 85 years of age more likely than adults 25 – 44 and 45 – 64 to have had an ED visit (24.9% vs 16.2% and 16.4%, respectively).

### Racial and Ethnic Group Differences in ED Use

**Figure 1:**
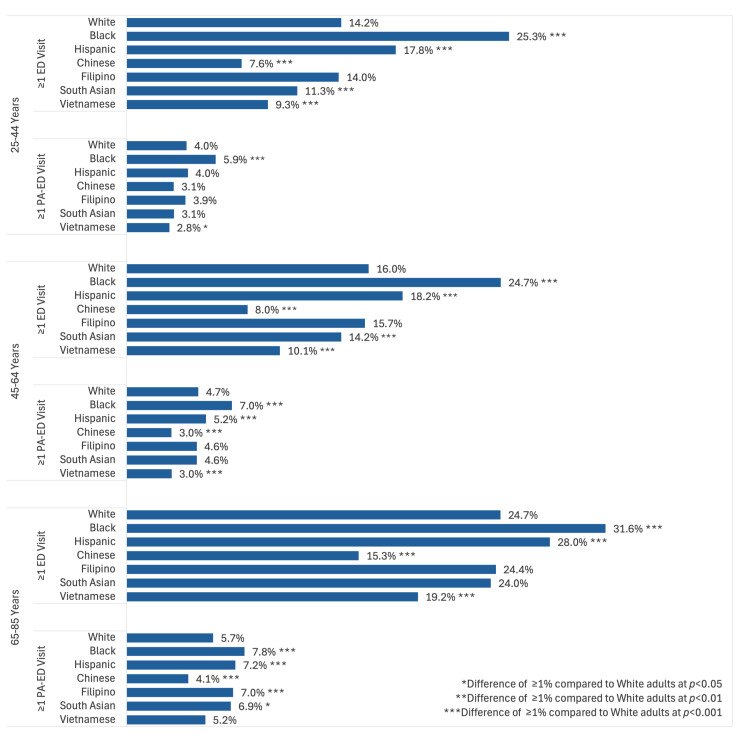
Percentages of adults with ≥ 1 emergency department visit in 2019 by race, ethnicity, and age group. *Racial/ethnic group significantly differs from White adults at *P* < .05.

**Figure 2:**
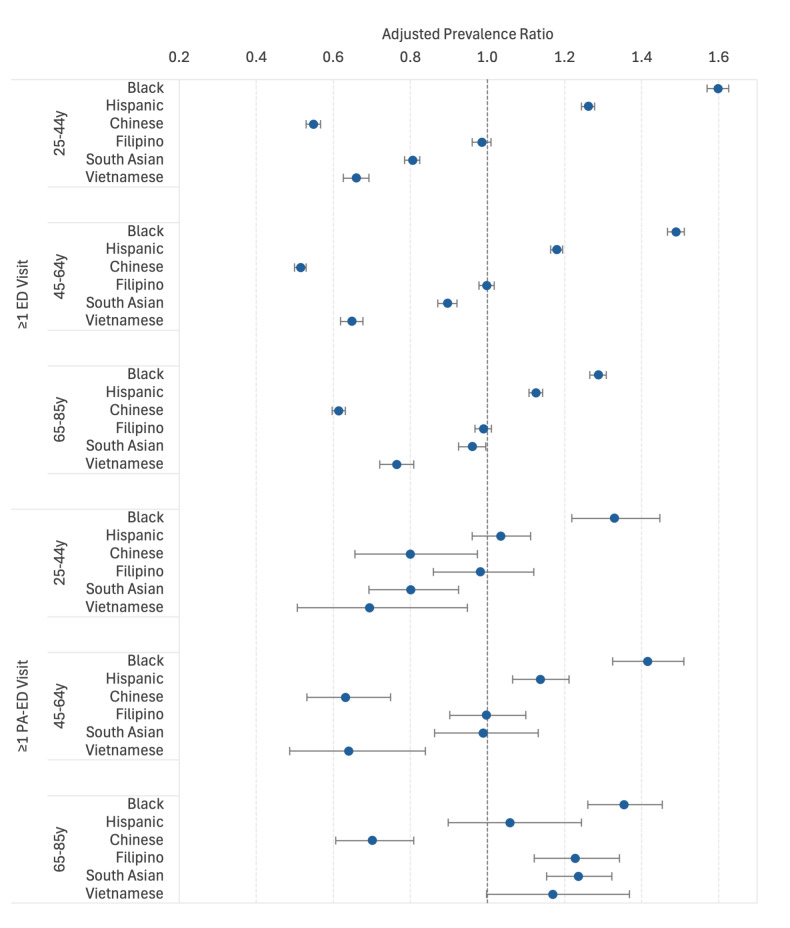
Adjusted prevalence ratios comparing prevalence of ≥ 1 emergency department visit among adults in six non-White racial and ethnic groups to White adults, by age group. Adjusted prevalence ratios with 95% confidence intervals compare prevalence of ≥ 1 emergency department visit among each non-White racial/ethnic group to prevalence among White adults in the same age group after adjusting for age as a 5-year interval variable, sex, non-English vs. English language preference, and Medicaid coverage status.

In all three age groups, Black and Hispanic adults were more likely and Chinese and Vietnamese adults less likely than White adults to have had an ED visit (Figure 1). South Asian adults were less likely than White adults to have had an ED visit in the 25 – 44 and 45 – 64 age groups, while Filipino adults did not meaningfully differ from White adults in any age group. The racial and ethnic group differences for ED visits remained significant after adjusting for sex, age, NELP status, and Medicaid coverage (Figure 2). Although the percentages of patients with only one ED visit were similar across racial/ethnic groups (≤ 0.5% for 25 – 44 years of age, ≤ 0.7% for 45 – 64, ≤ 1.3% for 65–85), the percentages of patients who had ≥ 5 ED visits varied widely, ranging from 4.3% for Chinese patients 25 – 44 years of age to 17.8% for Black patients. This pattern was consistent within all age groups, but the 65 – 85 years age group had higher numbers of ED visits overall. (Supplemental File 1).

### Sociodemographic and Medicaid Coverage Factors Associated with Having ≥ 1 ED Visit

#### Sex

**Figure 3:**
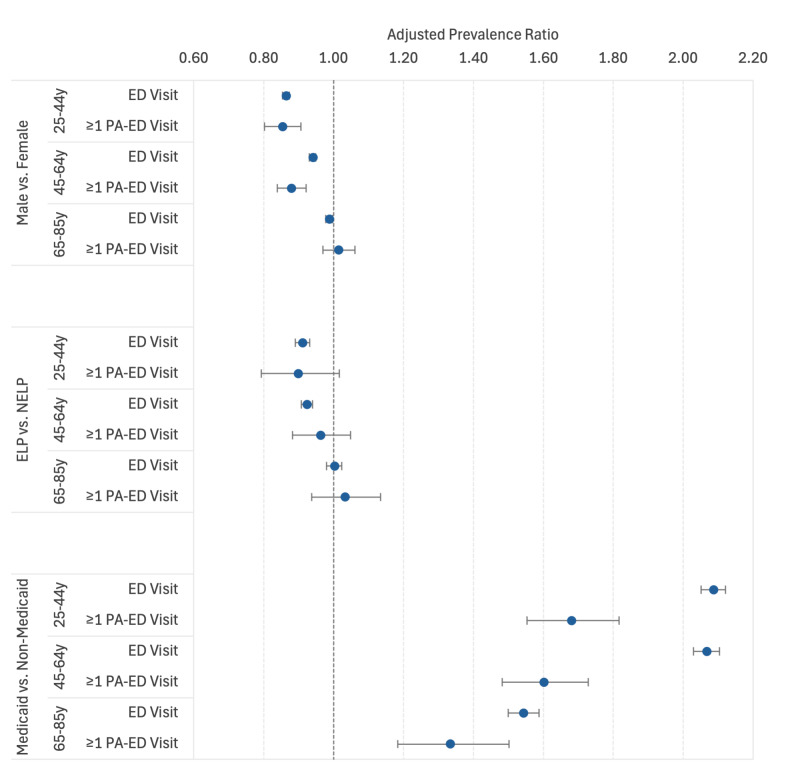
Adjusted prevalence ratios for ≥ 1 emergency department visit by sex, language preference, and Medicaid coverage status within three age groups. Adjusted prevalence ratios (with 95% confidence intervals) for ≥ 1 ED visit comparing males to females after adjusting for age as a 5-year interval variable, racial/ethnic group, language preference, and Medicaid status; non-English vs. English language preference after adjusting for age, sex, racial/ethnic group, and Medicaid status; and Medicaid to non-Medicaid covered adults after adjusting for age, sex, racial/ethnic group, and language preference. *ELP*, English language preference; *NELP*, non-English language preference.

In the full study population, after controlling for other covariates, males in the 25 – 44 and 45 – 64 age groups were less likely than similarly aged females to have had ≥ 1 ED visit (aPR = 0.86 [0.85 – 0.87] and aPR = 0.94 [0.93–0.95], respectively). However, no significant sex difference was seen for prevalence of ≥ 1 ED visit in the 65 – 85 age group (Figure 3). Within racial and ethnic groups, Black, Hispanic, and Filipino males in all age groups, White, Chinese, South Asian, and Vietnamese males in the 25 – 44 age group, and South Asian males in the 45 – 64 age group were less likely than females in the same age group to have had an ED visit (Supplemental File 2).

#### Non-English vs English Language Preference

In the full study population, after controlling for other covariates, NELP adults in the 25 – 44 and 45 – 64 age groups were approximately 10% less likely than their ELP counterparts to have had an ED visit (aPR = 0.91 [0.89 – 0.93] and aPR = 0.92 [0.91 – 0.94], respectively), but no association with NELP status was seen in the 65–85 group (Figure 3).

**Figure 4:**
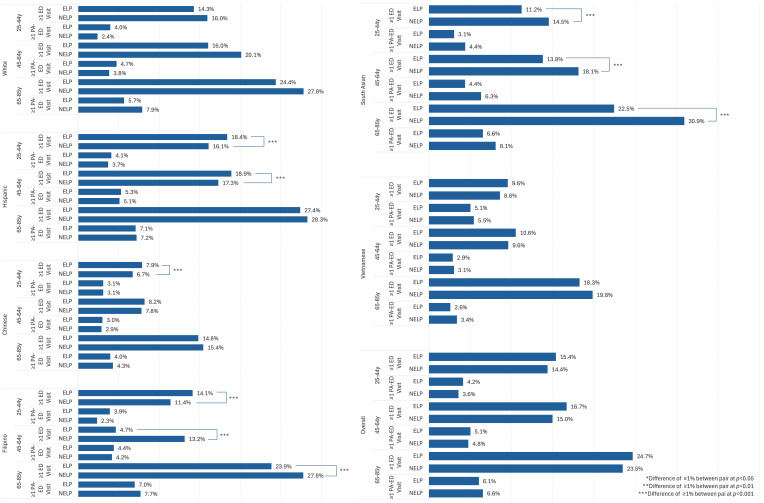
Prevalence of ≥ 1 emergency department visit by language preference, overall and within racial and ethnic groups. * NELP subgroup significantly differs from ELP subgroup *P* < .05. *NELP*, non-English language preference; *ELP*, English language preference.

Comparisons of the prevalence of ≥ 1 ED visit within racial and ethnic groups and prevalence of ≥ 1 ED visit by NELP vs ELP status within racial and ethnic groups are shown in Figure 4. Adjusted prevalence ratios comparing NELP to ELP subgroups within racial and ethnic groups are found in Supplemental File 2. Hispanic, Filipino, Chinese, and Vietnamese adults with NELP in the 25 – 44 and 45 – 64 age groups were 10 – 20% less likely to have had an ED visit than their ELP counterparts (aPR range: 0.80 – 0.91). However, White and South Asian adults with NELP in the 45 – 64 group and Filipino and South Asian adults with NELP in the 65 – 85 group were at least 10% more likely to have had an ED visit.

### Medicaid Coverage

In the full study population, after controlling for sociodemographic covariates, adults with Medicaid coverage were twice as likely as those not covered by Medicaid to have ≥ 1 ED visit in the 25 – 44 and 45 – 64 age groups (aPR = 2.09 [2.05 – 2.12] and aPR = 2.07 [2.03 – 2.10], respectively) and 50% more likely in the 65–85 age group (aPR = 1.54 [1.50 – 1.59]). Having Medicaid coverage approximately doubled the likelihood of having an ED visit in all racial and ethnic groups in the 25 – 44 and 4 5– 64 age groups (aPR range: 1.84 – 2.55), with a slightly lesser association in the 65–85 age group (aPR range: 1.23 – 1.89, but no significant difference among Vietnamese adults).

#### Outpatient Clinic Visit

In this health plan, an effort is made to connect all patients with a primary care doctor upon enrollment. Over 70% of adults 25 – 64 years of age and over 90% of adults 65 – 85 years of age had at least one outpatient clinic visit during 2019, with little variation across racial, ethnic, and language subgroups. However, clinic visits could not be temporally associated with ED visit use.

## DISCUSSION

Our aim in this study was to disaggregate the associations of race and ethnicity, language preference, and Medicaid coverage with the likelihood of having ≥ 1 ED in an insured adult population that was covered by the same health plan and received healthcare within the same integrated healthcare delivery system. We found that in this health plan population, Black and Hispanic adults were more likely than similarly aged White adults to have had ≥ 1 ED visit during 2019. These findings are consistent with previous population-based studies that included adults without health insurance and a regular source of primary care, which found that Black adults were more likely than White and Hispanic adults to have an ED visit,^16^–^18^ and that Black and Hispanic adults were more likely than White adults to make multiple ED visits and to use the ED for non-urgent care.^19^,^20^

We believe this is the first US study to examine ED use by different Asian ethnic subgroups, documenting a lower prevalence of ED use among Chinese, Vietnamese, and South Asian adults and similar prevalence among Filipino adults compared to White adults. A growing body of research points to the importance of using disaggregated data for Asian ethnic groups when studying health outcomes and healthcare utilization to improve health equity. For example, significant differences across US Asian ethnic groups have been documented in the prevalence of smoking, obesity, diabetes, hypertension, and coronary artery disease^13^,^21^ and in mortality from ischemic heart disease, heart failure, and stroke.^22^

With respect to language preference, adults 25 – 64 years of age with NELP were approximately 10% less likely than those with ELP to have had an ED visit. This is consistent with previous studies that found NELP status had little or no impact on likelihood of having an ED visit.^23^–^25^ However, we further showed that the impact of NELP status on ED use varied by racial and ethnic group within the three age strata, slightly lowering likelihood of ED use among Hispanic, Filipino, Chinese, and Vietnamese adults in the 25 – 44 and 45 – 64 age groups and slightly increasing likelihood among White and South Asian adults in the 45 – 64 age group and Filipino and South Asian adults in the 65 – 85 age group.

Several previous studies have reported substantially higher ED usage among adults covered by Medicaid as compared to private insurance.^26^–^29^ In our study, we also found that Medicaid coverage was associated with an approximately twofold higher prevalence of ED use among adults 25 – 64 years of age and a 50% higher likelihood among adults 65 – 85 across all racial/ethnic groups. This occurred even though all members of the study population had a usual source of primary and specialty care with access to limited evening and weekend outpatient care and a 24/7 advice nurse line, an assigned primary care physician and, for selected chronic health conditions, a care manager. We were unable to examine whether the higher ED usage among Medicaid-covered members was associated with a higher disease burden or social factors such as work- or childcare-related barriers. However, we do know Medicaid-covered members had no financial disincentive for ED use (ie, no copay), while non-Medicaid covered members, including those covered by Medicare, had a substantially higher co-pay for ED visits that did not result in a hospitalization. Higher ED visit copays have been shown to decrease likelihood of ED use for non-urgent reasons.^29^–^31^

## LIMITATIONS

This study had several limitations. The study data were for insured adults who all received care from the same integrated healthcare delivery system and thus may not be generalizable to populations that include uninsured adults, adults with more fragmented sources of healthcare, and adults without a primary care physician. Additionally, because this was a secondary analysis of an existing dataset, we lacked data to control for health burden and education, income, and other social determinants of health when comparing ED use by race and ethnicity, language preference, and Medicaid coverage status. Our dataset only captured ED visits within KPNC; so patients who presented to outside EDs would not have been captured. While KPNC covers out-of-plan ED visits for Medicaid and non-Medicaid covered members at the same copay level as the member would have for an in-plan ED visits, our study lacked data to evaluate whether out-of-plan ED use differed by Medicaid coverage status or other demographic factors. The dataset also lacked detailed information about the reason for the ED visit (eg, whether it was considered a true emergency), whether the individual was told to go to the ED by health plan staff, and whether the visit occurred outside regular outpatient clinic hours.

Furthermore, EHR data for race and ethnicity and language preference may contain some inaccuracies. Specifically, some adults with Asian race in the EHR but not Asian ethnicity were assigned to an Asian ethnic group based on surname, and some individuals who reported themselves as White but had first and last names and/or a spoken or written language preference that indicated they were likely South Asian, Hispanic/Latino, or Middle Eastern were re-assigned to a different racial/ethnic group. Additionally, at the time the source dataset was created, the health plan’s EHR only captured one racial/ethnic value for each member based on the US Office of Management and Budget’s six category combined race and ethnicity question. This, plus the use of a study-created algorithm to assign individuals to one racial/ethnic group when data for that individual from multiple sources indicated potential mixed race or ethnicity, may have led to misclassification or confounding due to mixed race/ethnicity in some instances.^13^

Further, although the source dataset allowed us to disaggregate Asian ethnic subgroups, we lacked the ability to do the same for the Black, White, and Hispanic groups. Finally, EHR-documented spoken language preference may not reflect a patient’s ability to communicate in English. For example, some individuals with a non-English language preference may have had limited English proficiency but some ability to communicate in English, and some with an English preference may not have communicated very well in English.

## CONCLUSION

In an adult health plan population that received care from the same integrated healthcare delivery system, we found substantial variation in prevalence of ED use by race and ethnicity, minimal difference by language preference, and substantially higher prevalence among adults with Medicaid coverage. Our study showed that differences between population subgroups can be masked when factors such as race, language status, and type of insurance coverage are simply controlled for in statistical models rather than examined using disaggregated data. Future studies should control for comorbidities associated with ED use, as well as social determinants of health such as educational attainment, access to food and housing, and environmental risks when comparing ED use across racial and ethnic groups as well as by language preference and Medicaid coverage status. To improve monitoring and to develop interventions to reduce ED visits, further research is needed to better understand the cultural and societal factors that drive ED usage in different segments of the population.

## Supplementary Information





## Figures and Tables

**Table:** Characteristics of the study population, overall and by racial and ethnic group.

Study subgroup	N	Percentage of total population	Mean age (Years)	Female sex	Non-English language preference	Covered by Medicaid
25–44 years of age						
Overall population	401,113	100.0%	35.4	53.0%	8.0%	4.7%
White	304,441	43.1%	35.3	51.2%	0.4%	3.9%
Black	50,715	7.2%	35.3	58.2%	0.5%	13.3%
Hispanic/Latino	191,949	27.2%	35.2	51.6%	22.0%	4.9%
Chinese	43,444	6.2%	35.7	57.5%	16.8%	3.0%
Filipino	43,943	6.2%	36	59.5%	1.7%	3.2%
South Asian	55,875	7.9%	35.3	52.1%	3.4%	2.9%
Vietnamese	15,187	2.2%	36.3	60.8%	18.1%	4.5%
45–64 years of age						
Overall population	410,724	100.0%	54.6	52.2%	11.9%	2.9%
White	433,903	51.4%	55.2	51.5%	0.6%	2.3%
Black	67,823	8.0%	54.6	55.8%	0.4%	6.0%
Hispanic/Latino	177,334	21.0%	53.5	50.9%	37.1%	3.2%
Chinese	57,459	6.8%	54.5	55.9%	34.3%	2.9%
Filipino	58,582	6.9%	54.3	57.6%	4.4%	1.7%
South Asian	32,256	3.8%	53	46.8%	9.8%	4.2%
Vietnamese	17,270	2.0%	53.2	49.8%	37.6%	5.2%
65–85 years of age						
Overall population	496,924	100.0%	72.5	55.2%	7.8%	1.8%
White	322,722	64.9%	72.6	54.8%	0.5%	0.7%
Black	35,279	7.1%	72.3	59.1%	0.3%	2.8%
Hispanic/Latino	61,748	12.4%	72.5	55.3%	28.8%	3.1%
Chinese	32,830	6.6%	72.3	53.4%	39.8%	4.4%
Filipino	29,722	6.0%	72.2	59.0%	6.6%	3.1%
South Asian	9,418	1.3%	71.8	46.1%	17.5%	10.1%
Vietnamese	5,205	1.0%	71.6	51.3%	53.7%	12.0%
