# Supplementary material for: Emergency Department Utilization by Race, Ethnicity, Language, and Medicaid Status
Source: West J Emerg Med. 2025 Jul 11;26(4):951–9. doi: 10.5811/westjem.41511 (PMC12342473; doi:10.5811/westjem.41511)
Supplement: Supplementary file 1 [file wjem-26-951-s001.docx]

**Supplemental Table 1. Total number of emergency department visits in 2019, by race and ethnicity and age group**

| **Age** | **# of ED** | **Race and ethnicity** | | | | | | | | | | | | | | | |
| --- | --- | --- | --- | --- | --- | --- | --- | --- | --- | --- | --- | --- | --- | --- | --- | --- | --- |
| **group** | **visits** | **White** | | **Black** | | **Hispanic** | | **Filipino** | | | **Chinese** | | | **South Asian** | | **Vietnamese** | |
| **25-44** |  | **n** | **%** | **n** | **%** | **n** | **%** | **n** | **%** | | **n** | | **%** | **n** | **%** | **n** | **%** |
|  | 0 | 261308 | 85.8% | 37906 | 74.7% | 157830 | 82.2% | 37788 | 86.0% | | 40141 | | 92.4% | 49583 | 88.7% | 13770 | 90.7% |
|  | 1 | 1372 | 0.5% | 291 | 0.6% | 959 | 0.5% | 198 | 0.5% | | 120 | | 0.3% | 191 | 0.3% | 60 | 0.4% |
|  | 2 | 3948 | 1.3% | 1062 | 2.1% | 3423 | 1.8% | 611 | 1.4% | | 441 | | 1.0% | 718 | 1.3% | 161 | 1.1% |
|  | 3-4 | 9989 | 3.3% | 2430 | 4.8% | 7672 | 4.0% | 1401 | 3.2% | | 866 | | 2.0% | 1487 | 2.7% | 377 | 2.5% |
|  | ≥ 5 | 27824 | 9.1% | 9026 | 17.8% | 22065 | 11.5% | 3945 | 9.0% | | 1876 | | 4.3% | 3896 | 7.0% | 819 | 5.4% |
| **45-64** |  | **n** | **%** | **n** | **%** | **n** | **%** | **n** | **%** | | **n** | | **%** | **n** | **%** | **n** | **%** |
|  | 0 | 364538 | 84.0% | 51066 | 75.3% | 145004 | 81.8% | 49364 | 84.3% | | 52869 | | 92.0% | 27686 | 85.8% | 15521 | 89.9% |
|  | 1 | 3039 | 0.7% | 503 | 0.7% | 1116 | 0.6% | 371 | 0.6% | | 225 | | 0.4% | 181 | 0.6% | 95 | 0.6% |
|  | 2 | 5051 | 1.2% | 1076 | 1.6% | 2460 | 1.4% | 687 | 1.2% | | 452 | | 0.8% | 332 | 1.0% | 145 | 0.8% |
|  | 3-4 | 11984 | 2.8% | 2298 | 3.4% | 5439 | 3.1% | 1541 | 2.6% | | 903 | | 1.6% | 776 | 2.4% | 337 | 2.0% |
|  | ≥ 5 | 49291 | 11.4% | 12880 | 19.0% | 23315 | 13.1% | 6619 | 11.3% | | 3010 | | 5.2% | 3281 | 10.2% | 1172 | 6.8% |
| **65-85** |  | **n** | **%** | **n** | **%** | **n** | **%** | **n** | **%** | | **n** | | **%** | **n** | **%** | **n** | **%** |
|  | 0 | 243065 | 75.3% | 24123 | 68.4% | 44488 | 72.0% | 22477 | 75.6% | | 27800 | | 84.7% | 7153 | 76.0% | 4204 | 80.8% |
|  | 1 | 4236 | 1.3% | 432 | 1.2% | 724 | 1.2% | 326 | 1.1% | | 328 | | 1.0% | 121 | 1.3% | 61 | 1.2% |
|  | 2 | 4794 | 1.5% | 592 | 1.7% | 899 | 1.5% | 429 | 1.4% | | 368 | | 1.1% | 151 | 1.6% | 71 | 1.4% |
|  | 3-4 | 9474 | 2.9% | 1069 | 3.0% | 1818 | 2.9% | 773 | 2.6% | | 565 | | 1.7% | 227 | 2.4% | 98 | 1.9% |
|  | ≥ 5 | 61153 | 18.9% | 9063 | 25.7% | 13819 | 22.4% | 5717 | 19.2% | | 3769 | | 11.5% | 1766 | 18.8% | 771 | 14.8% |
|  | | | | | | | | | |  | |  |  |  |  |  |  |
