# Supplementary material for: Emergency Department Utilization by Race, Ethnicity, Language, and Medicaid Status
Source: West J Emerg Med. 2025 Jul 11;26(4):951–9. doi: 10.5811/westjem.41511 (PMC12342473; doi:10.5811/westjem.41511)
Supplement: Supplementary file 2 [file wjem-26-951-s002.docx]

**Supplemental file 2. Comparison of prevalence of ≥ 1 emergency department visit by sex, Medicaid coverage status, and language preference, adjusting for covariates, by racial and ethnic group within 3 age groups**

| **Race/ethnicity** | **Comparison** |  | **Ages 25-44** | | **Ages 45-64** | | **Ages 65-85** | |
| --- | --- | --- | --- | --- | --- | --- | --- | --- |
|  |  | | **aPR** | **95% CI** | **aPR** | **95% CI** | **aPR** | **95% CI** |
| **White** | **Male vs. female** | | **0.90** | **[0.88-0.91]** | **0.99** | **[0.98-1.01]** | **1.01** | **[1.00-1.02]** |
|  | **Medicaid vs. non-Medicaid** | | **2.22** | **[2.16-2.29]** | **2.07** | **[2.01-2.13]** | **1.89** | **[1.80-1.98]** |
|  | **NELP vs. ELP** | | **1.02** | **[0.90-1.16]** | **1.16** | **[1.08-1.25]** | **1.00** | **[0.92-1.09]** |
| **Black** | **Male vs. female** | | **0.78** | **[0.76-0.81]** | **0.92** | **[0.90-0.94]** | **0.96** | **[0.93-0.99]** |
|  | **Medicaid vs. non-Medicaid** | | **2.01** | **[1.95-2.08]** | **2.11** | **[2.04-2.18]** | **1.68** | **[1.57-1.79]** |
|  | **NELP vs. ELP** | | **0.69** | **[0.52-0.91]** | **0.78** | **[0.62-0.98]** | **0.83** | **[0.63-1.11]** |
| **Hispanic** | **Male vs. female** | | **0.85** | **[0.83-0.86]** | **0.85** | **[0.83-0.86]** | **0.91** | **[0.88-0.93]** |
|  | **Medicaid vs. non-Medicaid** | | **1.96** | **[1.91-2.02]** | **2.01** | **[1.94-2.08]** | **1.49** | **[1.40-1.57]** |
|  | **NELP vs. ELP** | | **0.91** | **[0.89-0.94]** | **0.91** | **[0.90-0.93]** | **1.00** | **[0.97-1.03]** |
| **Filipino** | **Male vs. female** | | **0.89** | **[0.85-0.94]** | **0.91** | **[0.88-0.95]** | **0.96** | **[0.92-1.00]** |
|  | **Medicaid vs. non-Medicaid** | | **1.90** | **[1.74-2.09]** | **1.75** | **[1.58-1.94]** | **1.23** | **[1.12-1.36]** |
|  | **NELP vs. ELP** | | **0.80** | **[0.65-0.98]** | **0.81** | **[0.73-0.90]** | **1.10** | **[1.02-1.18]** |
| **Chinese** | **Male vs. female** | | **0.87** | **[0.82-0.94]** | **0.99** | **[0.93-1.05]** | **1.02** | **[0.97-1.07]** |
|  | **Medicaid vs. non-Medicaid** | | **1.90** | **[1.65-2.18]** | **2.04** | **[1.81-2.29]** | **1.32** | **[1.18-1.47]** |
|  | **NELP vs. ELP** | | **0.83** | **[0.75-0.91]** | **0.90** | **[0.85-0.95]** | **0.96** | **[0.91-1.01]** |
| **South Asian** | **Male vs. female** | | **0.83** | **[0.79-0.87]** | **0.92** | **[0.87-0.97]** | **1.05** | **[0.98-1.13]** |
|  | **Medicaid vs. non-Medicaid** | | **2.55** | **[2.34-2.76]** | **2.20** | **[2.02-2.40]** | **1.28** | **[1.15-1.43]** |
|  | **NELP vs. ELP** | | **1.09** | **[0.98-1.23]** | **1.11** | **[1.02-1.20]** | **1.27** | **[1.16-1.39]** |
| **Vietnamese** | **Male vs. female** | | **0.90** | **[0.81-1.00]** | **0.95** | **[0.86-1.03]** | **0.96** | **[0.85-1.07]** |
|  | **Medicaid vs. non-Medicaid** | | **1.96** | **[1.65-2.33]** | **1.84** | **[1.58-2.14]** | **1.09** | **[0.92-1.28]** |
|  | **NELP vs. ELP** | | **0.86** | **[0.75-0.99]** | **0.86** | **[0.78-0.94]** | **1.02** | **[0.91-1.15]** |
| **aPR: Adjusted prevalence ratio; CI: Confidence interval; NELP: Non-English language preference; ELP: English language preference. APRs are estimated from models that control for sex, age (as a 5-year interval variable), NELP/ELP status, and Medicaid coverage. Bolded aPRs indicate a ≥ 10% difference in prevalence between the subgroups.** | | | | | | | | |
